# V-shaped double-row distal triceps tendon repair: a novel technique using unicortical button fixation

**DOI:** 10.1186/s40001-017-0250-4

**Published:** 2017-03-14

**Authors:** Bastian Scheiderer, Lucca Lacheta, Andreas B. Imhoff, Sebastian Siebenlist

**Affiliations:** Department of Orthopedic Sports Medicine, Klinikum rechts der Isar, Technical University of Munich, Ismaninger Strasse 22, 81675 Munich, Germany

**Keywords:** Distal triceps, Double row, unicortical button, Tendon repair

## Abstract

**Background:**

This report was designed to present a novel technique combining suture anchor and unicortical button fixation for distal triceps tendon repair.

**Technical description:**

For anatomical reinsertion of an acute distal triceps tendon rupture, two suture anchors and one unicortical button forming a V-shaped configuration were used. The operative approach is described in detail.

**Results:**

Excellent clinical and functional results were achieved in the early postoperative phase. The patient reached full elbow range of motion and extension muscle strength (5/5) compared to the uninjured arm 12 weeks after surgery. Complications did not occur.

**Conclusion:**

This is the first report using unicortical button fixation in distal triceps tendon repair with promising preliminary results.

**Level of evidence:**

Technical description, case report, Level V

## Background

The distal triceps tendon rupture is a rare entity [1–3]. Anzel et al. published a series of 1014 cases of tendon and muscle disruption. Only 1% of them involved the distal triceps tendon [4]. The mechanism of injury is mainly a fall on the outstretched forearm or contraction against resistance (weightlifting) [5, 6]. Triceps tendon ruptures are furthermore associated with systemic diseases (e.g. hyperparathyroidism), corticosteroid use and anabolic steroid abuse [7, 8]. Especially men and professional American football players are at increased risk to suffer distal triceps tendon tears [6]. Anatomic studies have shown that total and/or partial tendon tears mostly occur at the bone–tendon interface on the olecranon insertion site, and also musculotendinous and intramuscular tears have been described [9–12].

For anatomic reconstruction of distal triceps tendon ruptures, a standard surgical technique has not yet been established. In the transosseous cruciate repair technique, Krakow-type sutures placed in the tendon are passed through two crossing bone tunnels and tied over a bone bridge. However, this procedure has shown a high re-rupture rate of up to 21% [13].

The purpose of currently published techniques is the anatomic reinsertion at the bony footprint of the olecranon with restored tendon–surface contact [14]. Learning from the progress of arthroscopic tendon repair at the shoulder joint, Yeh et al. reported of a suture anchor repair for distal triceps ruptures [15]. The authors compared this single- and double-row suture anchor repair technique with the transosseous cruciate repair technique. They could demonstrate that the anatomic repair with a suture bridge consisting of four anchors provides superior footprint contact characteristics and lowest displacement during increased cyclic loading compared to single-row repair and transosseous cruciate repair [15]. In 2014, Clark et al. reported on a new knotless anatomic repair technique to minimise the risk of intra-articular joint breach, knot failure, as well as bursal and subcutaneous irritation. Two bone tunnels and one knotless anchor were used. This technique showed significantly higher load and cycle to failure compared to the traditional transosseous cruciate repair [16, 17].

In the present article, we describe a novel technique for anatomic footprint repair in case of distal triceps tendon ruptures using two suture anchors and one intramedullary placed button in a V-shaped double-row configuration.

## Case presentation and technical description

We report on the case of a 30-year-old male patient with an acute distal triceps tendon rupture after a snowboarding accident. The patient described a fall onto the extended forearm followed by immediate pain and weakness upon extension of the elbow. Clinical evaluation showed an inability to extend the elbow against resistance. Radiographs of the elbow (ap- and lateral-view) excluded osseous lesion. Subsequent magnetic resonance imaging (MRI) confirmed the diagnosis of a total rupture of the triceps tendon with 15 mm of retraction.

For surgery (14 days after injury), the patient was positioned in the prone position. A standard posterior approach was used for exposure of the distal triceps tendon rupture. The tendon was mobilised and debrided at the rupture site. Then, the footprint at the olecranon was identified and the bone bed debrided. Two 5.5-mm double-loaded titanium suture anchors (5.5 Corkscrew FT, Arthrex Inc., Naples, FL, USA) were placed at the proximal area of the footprint, one medial and one lateral. A Krakow whipstitch was performed at the distal triceps tendon with both ends of each Fiberwire^®^. In the following, the sutures were tied. Subsequently, one end of each suture was cut.

**Fig. 1:**
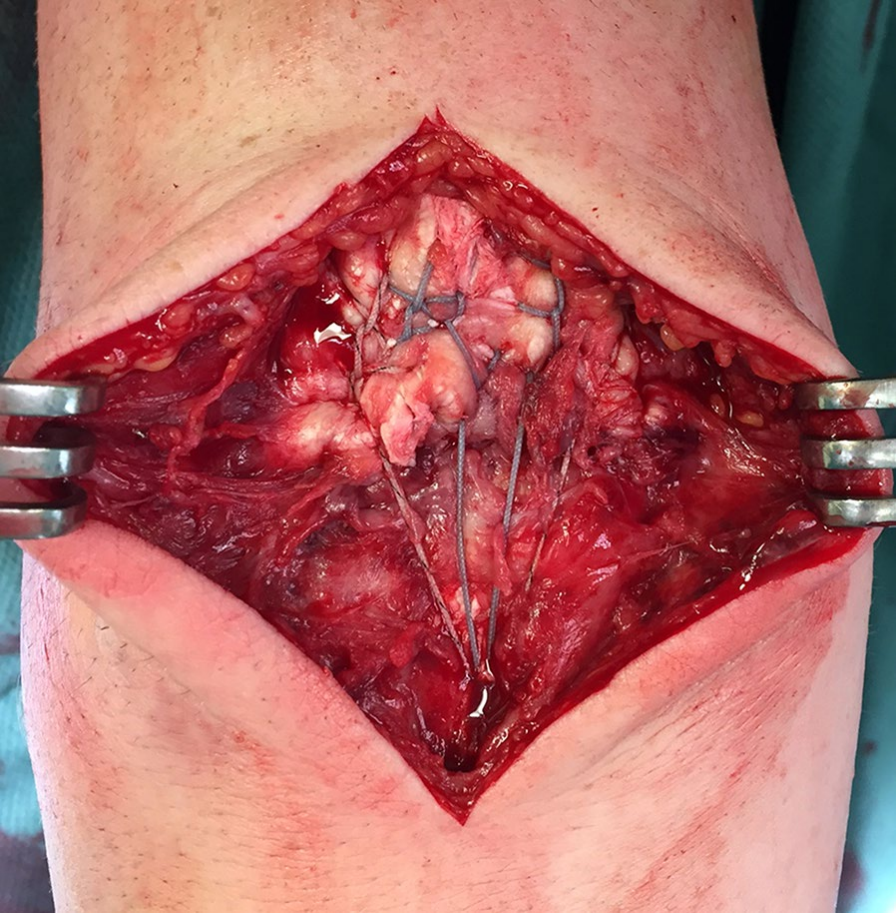
Intraoperative situs of the V-shaped technique: the unicortical fixation using a BicepsButton™ provides a planar contact pressure of the triceps tendon

**Fig. 2:**
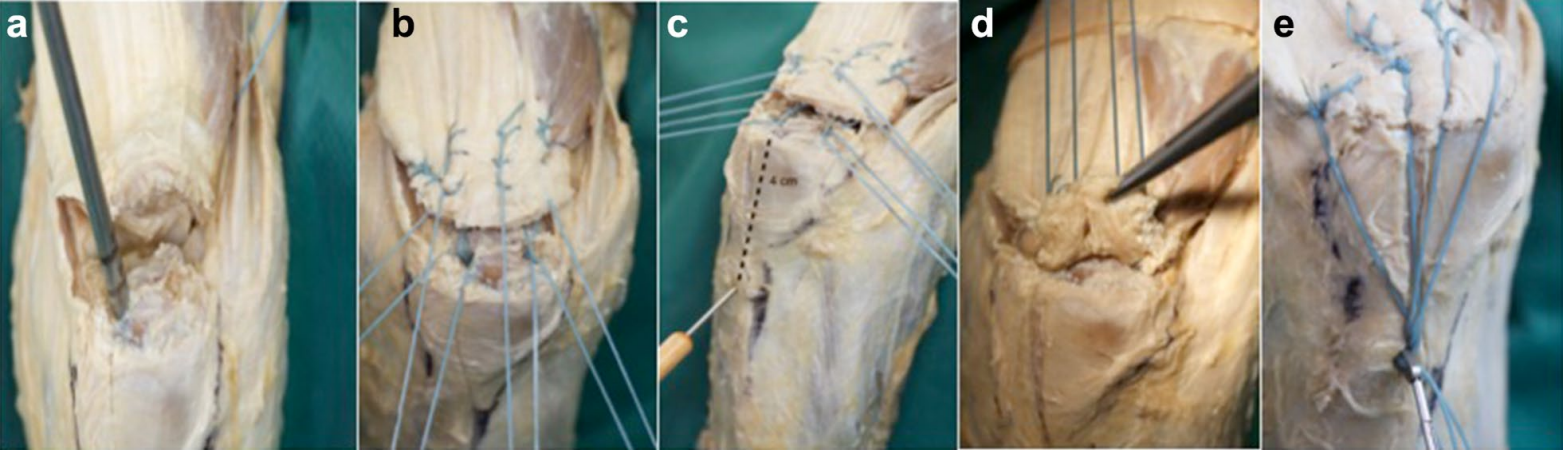
Cadavaric demonstration of the double-row V-shaped triceps repair. **a** Following debridement, pilot holes for two 5.5-mm suture anchors are created at the proximal border of the footprint. **b** Krakow whipstich sutures placed along the medial, lateral and central part of the triceps tendon. **c** 4 cm distal to the footprint line, a monocortical 3.2-mm drill-hole is placed in an angle of 45° in proximal direction to the ulnar shaft. **d** Result following knot tying creating a proximal row repair, subsequently one end of each suture is cut. **e** Loaded BicepsButton™. Before it is passed through the posterior cortex, the cancellous bone within the intramedullary canal should be compressed using a small clamp

In the next step, the V-shaped double-row fixation was performed using the unicortical button fixation technique analogous to the previously described distal biceps tendon repair [18]. Four centimetre distal to the distal footprint line, a 3.2-mm drill-hole was centrally drilled into the posterior cortex of the ulna at an angle of 45° (with proximal direction) related to the ulnar shaft. The cancellous bone within the intramedullary canal was then compressed using a small clamp to create space for the BicepsButton™ (Bicepsbutton, Arthrex Inc., Naples, FL, USA) implantation. Next, the button was loaded with all four FibreWire sutures (in reversed fashion), passed through the posterior cortex and flipped intramedullary (Fig. 1). Due to this V-shaped suture configuration, the distal tendon stump was planar pressed to its insertion. Each suture was strongly tightened after flipping the button to compress cancellous bone at the intramedullary canal. Like a pulley system principle, the tension onto the reconstructed distal triceps tendon footprint could then be modified for optimal tendon–bone pressure before knotting. The elbow was finally moved with full range of motion (ROM) conditioning the construct. If necessary, the V-shaped pulley system could have been retightened. Skin closure was performed in a standard manner. The detailed operative approach is demonstrated in Fig. 2.

For postoperative management, the elbow was immobilised in a posterior splint (90° of elbow flexion) for 5 days. Subsequently, a mobile, hinged brace (Epico ROM, medi, Bayreuth, Germany) was applied for 6 weeks, limiting elbow flexion to 90°. Passive and active (gravity-assisted) motion was started at day 1 after surgery with restriction of active extension for 6 weeks. Sports activities were allowed after 12 weeks.

**Fig. 3:**
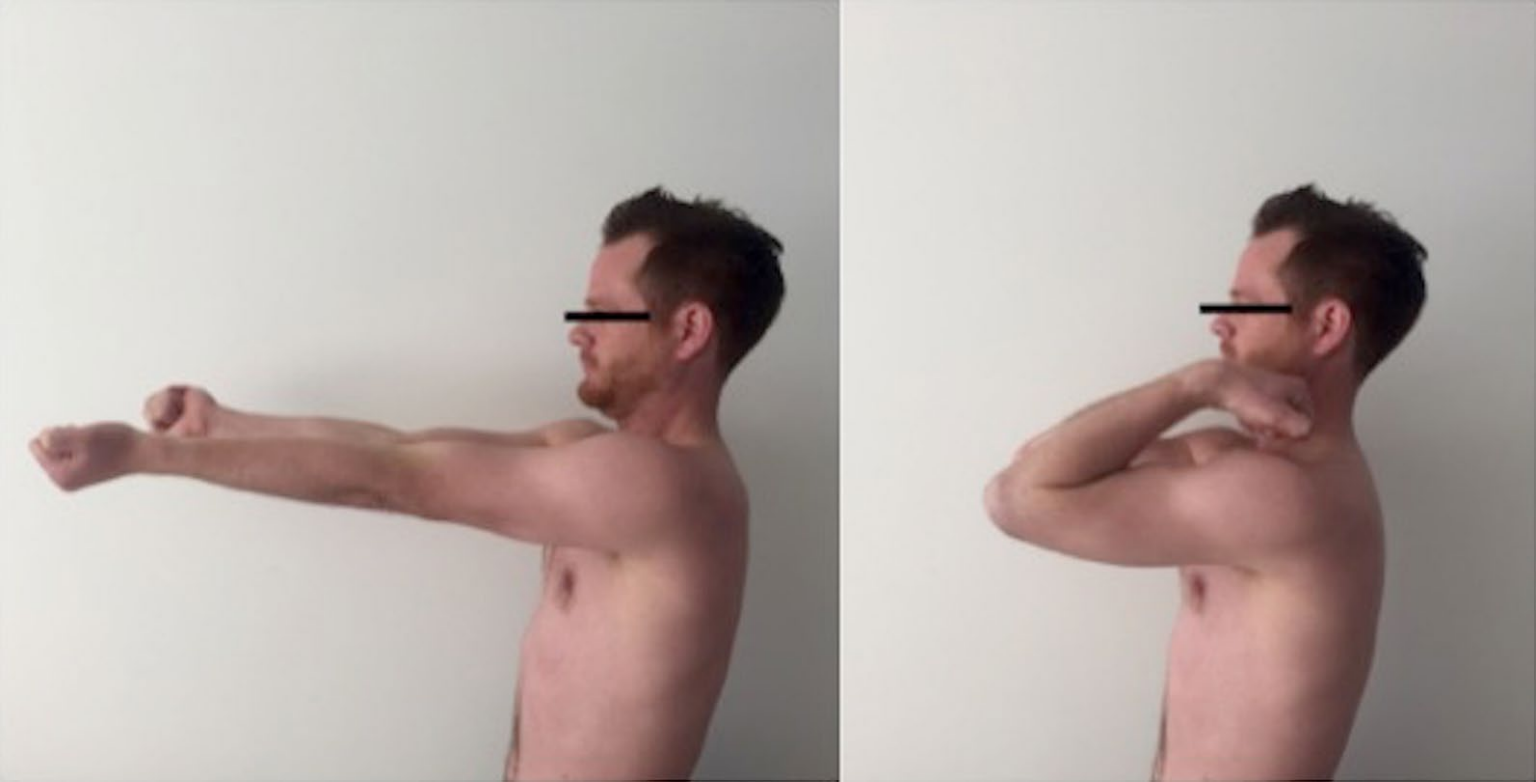
Full active ROM 12 weeks postoperatively

**Fig. 4:**
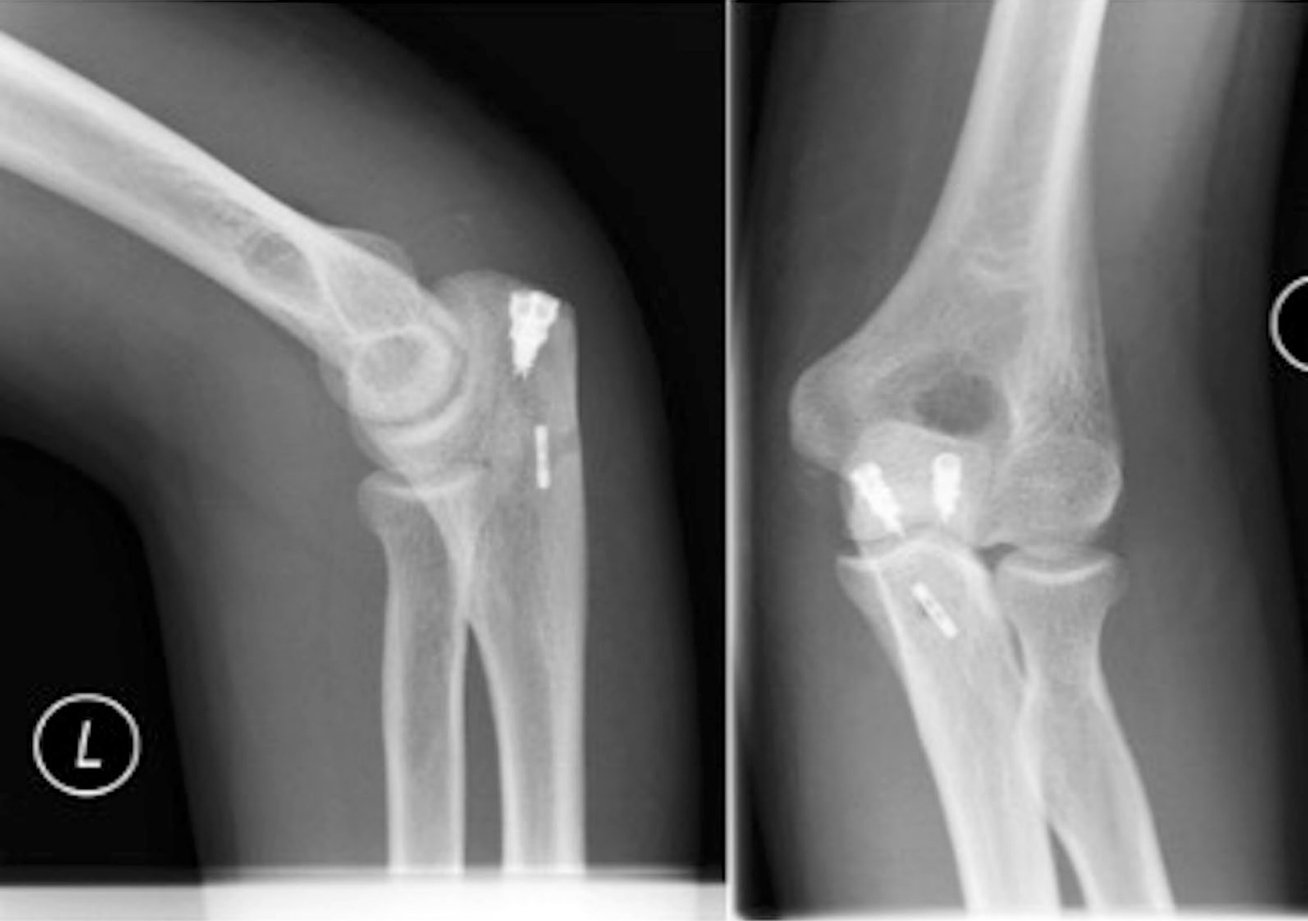
Postoperative radiograph showing the intramedullary cortical button

The patient was very satisfied and would undergo the same surgical procedure again. Follow-up examinations after 12 weeks showed full elbow range of motion (flexion/extension 130°–0°–0°) (Fig. 3). Comparable strength of elbow extension (muscle strength 5/5 according to Janda scale [19]) was measured in relation to the contralateral site at 12-week follow-up. Postoperative radiographs showed no implant displacement (Fig. 4).

## Discussion and conclusions

The novel technique of double-row distal triceps tendon repair performing a V-configuration by unicortical button fixation showed an excellent functional outcome in the present case. To the best of our knowledge, this is the first report dealing with unicortical button fixation in triceps tendon repair.

The biomechanical benefits of complete anatomical footprint coverage with double-row repair technique have already been demonstrated in multiple studies for rotator cuff repair [20–26].

Kim et al. showed superior results in strength, stiffness and gap formation using the double-row technique. Mazzocca et al. found equivalent results for load-to-failure, cyclic loading and gap formation for the single-row technique compared to the double-row repair. However, a larger area of the footprint was restored using the double-row technique [27, 28]. These biomechanical findings were now transferred to distal triceps tendon repair [15].

The V-shaped double-row repair technique combines the outclassing properties of an anatomical footprint coverage and technical advantage of using a single fixation distal to the joint. The unicortical button fixation enables a modification in tendon contact pressure by tightening or loosening the sutures, once the fixation system has been installed. This represents a technical key benefit compared to the suture bridge repair or the knotless anatomic repair. Similar to already-described techniques, an accelerated rehabilitation program is practicable due to double-row repair. Titanium suture anchors were used in the present case; however, bio-absorbable suture anchors would be a potential alternative for proximal row fixation.

Siebenlist et al. established “the intramedullary cortical button fixation technique” for distal biceps tendon repair [18, 29]. They have shown that biomechanical characteristics for this repair technique are comparable or superior to suture anchor repair and bicortical button fixation, respectively [18, 30]. Buchholz et al. also found no major differences between monocortical button fixation versus interference screw for subpectoral proximal biceps tenodesis [31]. However, it has to be clearly stated that no biomechanical studies exist for unicortical button fixation in distal triceps tendon repair.

The main advantages of the present fixation technique are the reduced risk of iatrogenic fractures at the proximal ulna due to monocortical drilling, a simple implantation and lower implant costs compared to double-row anchor systems (two anchors and one button instead of four anchors) [16, 17]. However, transosseous techniques have the favourable costs. Additionally, this technique facilitates a complete footprint coverage with a flat contact pressure to possibly favour tendon-to-bone healing. The preliminary excellent functional results in the present case are encouraging to continue using the V-shaped double-row repair technique for triceps tendon ruptures. Nevertheless, the present report has a limited clinical follow-up of 3 months what is too short to compare this technique to others. Therefore, further follow-up evaluation including objective strength measurements and MRI controls is mandatory.

The V-shaped double-row fixation represents a novel, alternative technique for treatment of distal triceps tendon ruptures with promising preliminary clinical results. Compared to previous published reconstruction methods, the advantage of this technique is the possible modification in tendon footprint coverage once the fixation system has been installed. Furthermore, the iatrogenic fracture risk can be reduced by monocortical drilling. However, additional studies are needed to evaluate the long-term efficacy of this surgical procedure.

## Abbreviations

ap: anterior–posterior
MRI: magnetic resonance imaging
ROM: range of motion
